# Urokinase Plasminogen Activator Induces Pro-Fibrotic/M2 Phenotype in Murine Cardiac Macrophages

**DOI:** 10.1371/journal.pone.0057837

**Published:** 2013-03-11

**Authors:** Jessica Meznarich, Laura Malchodi, Deri Helterline, Stephen A. Ramsey, Kate Bertko, Tabitha Plummer, Abigail Plawman, Elizabeth Gold, April Stempien-Otero

**Affiliations:** 1 University of Washington School of Medicine, Division of Cardiology, Seattle, Washington, United States of America; 2 Seattle Biomedical Research Institute, Seattle, Washington, United States of America; Albert Einstein College of Medicine, United States of America

## Abstract

**Objective:**

Inflammation and fibrosis are intertwined in multiple disease processes. We have previously found that over-expression of urokinase plasminogen activator in macrophages induces spontaneous macrophage accumulation and fibrosis specific to the heart in mice. Understanding the relationship between inflammation and fibrosis in the heart is critical to developing therapies for diverse myocardial diseases. Therefore, we sought to determine if uPA induces changes in macrophage function that promote cardiac collagen accumulation.

**Methods and Results:**

We analyzed the effect of the uPA transgene on expression of pro-inflammatory (M1) and pro-fibrotic (M2) genes and proteins in hearts and isolated macrophages of uPA overexpressing mice. We found that although there was elevation of the pro-inflammatory cytokine IL-6 in hearts of transgenic mice, IL-6 is not a major effector of uPA induced cardiac fibrosis. However, uPA expressing bone marrow-derived macrophages are polarized to express M2 genes in response to IL-4 stimulation, and these M2 genes are upregulated in uPA expressing macrophages following migration to the heart. In addition, while uPA expressing macrophages express a transcriptional profile that is seen in tumor–associated macrophages, these macrophages promote collagen expression in cardiac but not embryonic fibroblasts.

**Conclusions:**

Urokinase plasminogen activator induces an M2/profibrotic phenotype in macrophages that is fully expressed after migration of macrophages into the heart. Understanding the mechanisms by which uPA modulates macrophage function may reveal insights into diverse pathologic processes.

## Introduction

Cardiac fibrosis, the accumulation of excess extracellular collagen, contributes to the significant morbidity and mortality of heart disease. Fibrosis is found in human end-stage heart disease independent of etiology [1], and occurs in both areas of acute ischemic injury [2] and areas of myocardium subjected to chronic stress [2], [3]. Fibrosis inhibits both systolic [4] and diastolic [5] function and is associated with atrial and ventricular arrhythmias [6]–[8]. Finally, fibrosis is a barrier to effective incorporation of exogenous cell therapies and may inhibit endogenous regeneration in the heart [9].

Macrophages are commonly found in association with fibrosis in cardiac tissue from subjects with end-stage heart disease [1]. In addition, macrophage accumulation is an early feature in multiple animal models of cardiac injury that result in fibrosis [10]. Macrophages may promote fibrosis via several mechanisms dependent upon the activation state of the macrophage (for review see [11], [12]). M1 macrophages stimulated by Th1 cytokines such as interferon gamma or exogenous lipopolysaccharide (LPS) elaborate proinflammatory cytokines that augment recruitment of additional macrophages and proteinases to degrade tissue and allow for the emigration of activated-collagen producing-fibroblasts. M2 macrophages are generated by stimulation with Th2 cytokines such as IL-4. These macrophages can produce the fibrogenic cytokine TGF-β1 and also directly stimulate fibroblasts to produce collagen.

There is a substantial literature linking pro-inflammatory Th1 cytokines to cardiac fibrosis. Elevated levels of the pro-inflammatory cytokines TNF-α, IL-1β, and IL-6 are found in both the circulation and fibrotic cardiac tissue of humans with end-stage heart failure [13], [14]. Elevation of these cytokines can be reproduced in animal models of cardiac injury and furthermore, inhibition of TNF-α, IL-1β, or IL-6 in some models can attenuate collagen deposition[15]–[17]. However, in other models inhibition of cytokine signaling has deleterious or neutral effects on cardiac repair [18]. The role of Th2 cytokine signaling pathways in cardiac repair is not well defined. TGF-β1 has been examined in models of cardiac repair [19]–[21]. However, these studies demonstrate that TGF- β1 predominantly modulates cardiomyocyte hypertrophy and survival. Studies on the role of M2 macrophages in cardiac repair and fibrosis are rare. A single study associates M2 polarization with fibrosis in a mouse model of myocarditis [22].

We have shown that the macrophage-derived proteinase, urokinase plasminogen activator (uPA), promotes spontaneous fibrosis limited to the heart [23]. This phenotype is dependent on the presence of plasminogen, a zymogen converted to the active substrate plasmin by uPA [24]. Plasmin activity is increased in hearts of humans with end-stage heart disease and fibrosis. In animal models, plasmin activity is increased in response to acute injury [25] and absence of uPA or plasminogen inhibits cardiac collagen deposition in response to ischemia, hypertension and myocarditis[26]–[28]. We hypothesized that uPA driven expression of macrophage-specific cytokines is a critical mediator of uPA-induced cardiac fibrosis. Here we show that increased cardiac uPA is associated with upregulation of the pro-inflammatory, M1 cytokine IL-6; however, absence of IL-6 does not attenuate uPA-induced fibrosis. Instead, our data indicate that a unique TGF-β1–independent M2 macrophage phenotype may be an important regulator of fibrosis in the heart. In sum, these data point to the complex pathways by which inflammation regulates fibrosis in the heart and the mechanisms by which increased uPA activity may modulate macrophage phenotype.

## Methods

### Ethics Statement

All animal protocols were approved by the University of Washington Office of Animal Welfare (protocol #4199–01) and conform to the Guide for the Care and Use of Laboratory Animals published by the US National Institutes of Health. Anesthetic methods to minimize suffering as much as possible are detailed in the Methods.

### Experimental Animals

Generation of SR-uPA^+/0^ mice has been previously described [29]. In this paper all mice described are of the high expressing M87 line. The SR-uPA^+/o^ mice were backcrossed into the C57BL/6 background for at least 8 generations then bred with nontransgenic C57BL/6 littermates to obtain experimental and control mice. Some mice were bred with mice deficient in IL-6 (*Il6^−/−^*, also C57/Bl6, Jackson Labs) to generate IL-6^+/−^ mice with and without the SR-uPA transgene. Littermates were bred to generate experimental mice.

### Histologic Analyses

At appropriate time-points, mice were deeply anesthetized with intra-peritoneal ketamine (130 mg/kg) and xylazine (8.8 mg/kg). Anesthesia was deemed adequate when mice no longer responded to foot pinch. Hearts were excised, placed in PBS, transferred to PBS with 5% dextrose and 25 mmol/L KCl to produce cardiac arrest, weighed and then placed in sucrose formalin fixative. Hearts were sectioned into three pieces (base, midventricle and apex) and processed into a single paraffin block. Macrophages were detected with a rat monoclonal antibody (anti-Mac-3, clone M3/84, 2.5 µg/ml; PharMingen) and quantified as previously described. [30] Collagen accumulation was quantified by picrosirius red staining of a single section from the mid-ventricle of each heart as previously described. Quantification of cardiac macrophages and collagen was done by observers blinded to genotype and treatment.

### Cell Culture

#### Isolation of bone marrow derived macrophages

Bone marrow was collected and cultured with media high in GM-CSF as previously described [24].

#### Isolation of macrophages from hearts of SR-uPA^+/0^ mice

Hearts were excised, washed in warmed HBSS to remove blood, minced, placed in warm digestion buffer containing Liberase TH (Roche, 5 mg/ml) and DNase1 (2000 units) for 5 minutes, triturated with a pipet and passed through a 40 um filter into ice cold stopping buffer containing 5 ml of 10% FBS. This process was repeated with the tissue remaining in the filter for a total of 5 digestions. Cells were centrifuged, supernatant discarded and red blood cells lysed with ACK lysing buffer (Gibco). Cells were then washed twice with Hanks Balanced Salt Solution (HBSS). To isolate macrophages from this mononuclear, myocyte-free cell population, cells were resuspended in 2 ml of HBSS, and incubated with 50 ul of anti-CD45 magnetic beads for 30 minutes (Miltenyi). Cells were then allowed to flow through the MACS magnetic column with magnet in place. After washing with MACS buffer, column was removed from the magnet and CD45+ cells washed out for collection. RNA was immediately collected from these cells using RNeasy kit. We have previously shown that the majority of CD45+ cells are Mac-3 positive in tissue sections of SR-uPA mice at several age points [23].

#### Isolation and culture of fibroblasts from hearts of wild-type mice

The mononuclear, myocyte-free cell population was isolated as described above. Of note, all tubes were treated with Sigmacote to prevent adherence of fibroblasts to the plastic tubes during isolation. After isolation, cells were plated in fibroblast growth medium (1∶1 DMEM:F12, 10% FBS, pen-strep, L-glut). Cells were washed and medium replaced on Day 1. On day 4, cells were about 80% confluent and medium exchanged for macrophage conditioned media as described below.

### Luminex Assay

Conditioned media was collected from bone marrow derived macrophages or cardiac explant cultures as previously described [24]. Media was incubated with beads from a mouse 20-plex cytokine detection kit (Invitrogen) in a 96 well plate. After addition of secondary antibodies, bound cytokine concentrations were measured using a Luminex plate reader. Standard curves were run for all cytokines and in the case of explant tissue media, values normalized to tissue weight.

### Taqman Quantitative PCR

RNA was isolated using RNAeasy (Qiagen). RNA was processed using the Thermoscientific Verso 1 stem RT-qPCR kit according to the manufacturer’s instructions. Pre-validated primer/probe sets were obtained from Applied Biosystems (Inventoried TaqMan® Gene Expression Assays: cat# 4331182). All PCR reactions were normalized internally using either an eukaryotic 18 S RNA primer set (Applied Biosystems; cat# 4319413E) or GAPDH RNA primer set as an endogenous control and run in triplicate. Quantitative Taqman PCR was performed using the Applied Biosystem 7900HT Real Time PCR System and analyzed using the Applied Biosystem 7900HT System sequence detection software, version 2.3.

### Statistical Analysis

Data that are normally distributed are presented as bar graphs with mean ± S.D. and are compared with Student’s t-test. Data that were not normally distributed are presented as dot plots with median (25–75% range). Group medians are compared with the Mann-Whitney rank-sum test. Some data not normally distributed is presented in figures with mean ± S.E.M. for clarity.

## Results

### The pro-inflammatory Cytokine IL-6 is Elevated but does not Modulate Cardiac Fibrosis in SR-uPA^+/0^ Mice

Because increased pro-inflammatory cytokines are associated with cardiac dysfunction and fibrosis, we tested the hypothesis that SR-uPA^+/0^ macrophages express excess levels of these cytokines. To minimize the effects of potential differences from *in vivo* cytokine levels between genotypes, we performed these studies on macrophages derived from *ex vivo* bone marrow culture from nontransgenic (NTG) and SR-uPA^+/0^ littermates. We measured cytokines in macrophage-conditioned media from unstimulated and LPS stimulated macrophages plated at equivalent densities. We detected no differences in measured signals for 20 cytokines, chemokines and growth factors (TNF-α, IFN-γ, IL-1α, -1β, -2, -4, -5, -6, -10, -12, -13, -17; CCL2 and 3, CXCL1, 9, and 10; GM-CSF, VEGF, FGFb) using the Luminex cytofluorometric bead assay in unstimulated (n = 5–6 mice per genotype) or LPS-stimulated (n = 3 separate mice per genotype) macrophage conditioned media.

**Figure 1:**
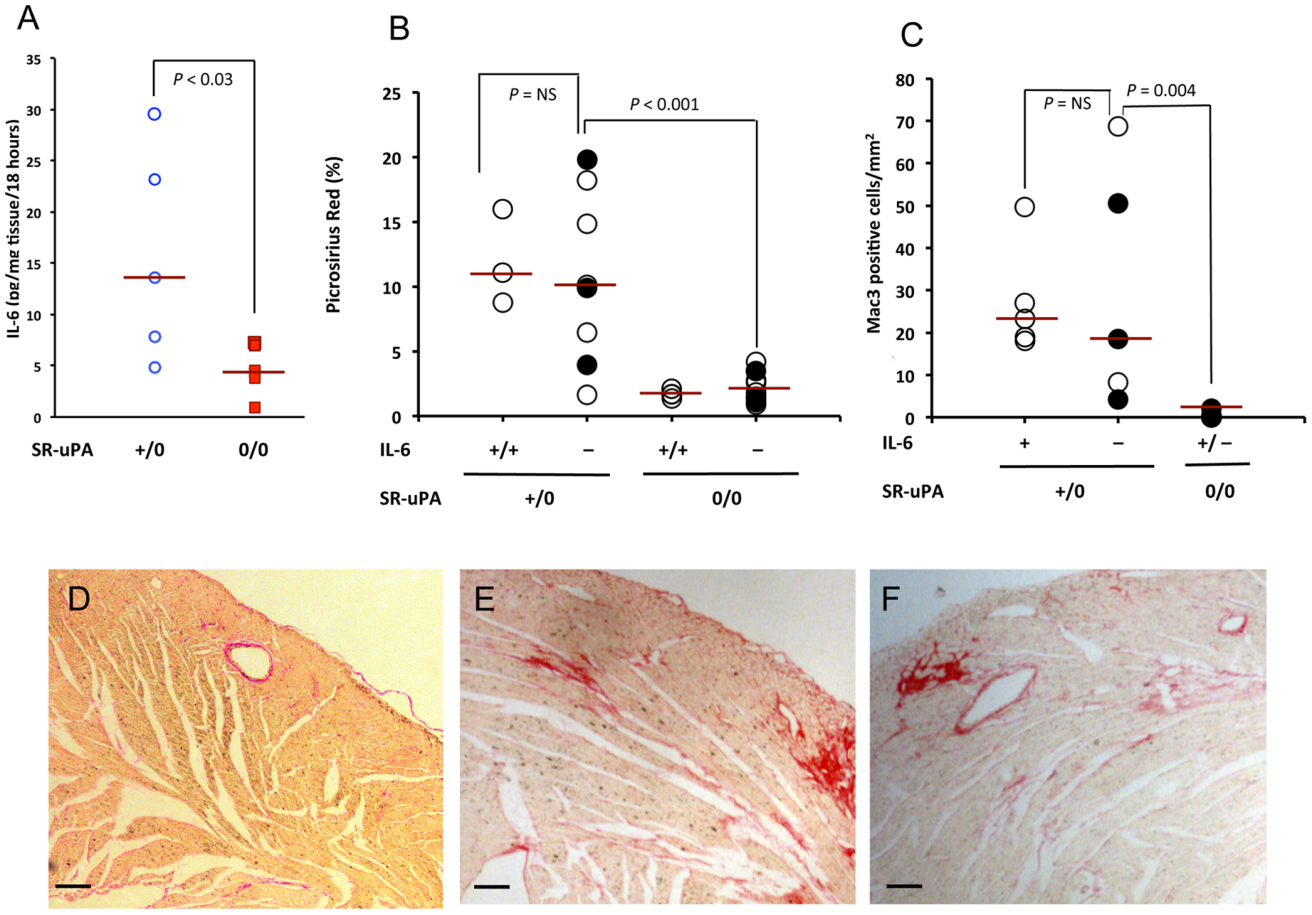
Cardiac IL-6 is elevated but does not mediate uPA-induced cardiac fibrosis. **A.** IL-6 concentrations in explant culture media from hearts of SR-uPA^+/0^ and NTG mice. N  = 5 mice per genotype. **B.** Collagen content of hearts from SR-uPA^+/0^ and NTG littermates at 15 weeks of age. Solid circles represent mice heterozygous for IL-6; open circles are mice homozygous (+/+ or −/−) for IL-6. Bars are medians. N  = 3–5 mice per heterozygous or homozygous genotype. **C.** Macrophage accumulation in hearts of SR-uPA^+/0^ and NTG littermates at 7 weeks of age. Solid circles represent mice heterozygous for IL-6; open circles are mice homozygous (+/+ or −/−) for IL-6. Bars are medians. N  = 3–5 mice per heterozygous or homozygous genotype. Representative heart sections stained with picrosirius red of **D.** NTG mice, **E.** SR-uPA^+/0^ *il6* ^+/+^ and **F.** SR-uPA^+/0^ *il6* ^−/−^ mice. Bars represent 100 µm.

To determine if macrophage specific over-expression of uPA increased expression of cytokines in cardiac tissue, we used the same assay to measure cytokine production from cardiac tissue explant cultures. Cardiac tissue was collected from 5–7 week old mice, a time-point at which there is robust accumulation of cardiac macrophages, but no fibrosis, in SR-uPA^+/0^ mice. All measurements were converted to picogram concentrations using standard curves generated for each cytokine and normalized to the tissue weight of the cultured tissue sample. Seven of 20 measured cytokines were measurable above the limits of detection of the assay (VEGF, CCL2, GM-CSF, IL-2, FGFb, KC, IL-6). Levels of IL-6 were significantly elevated in explant-cultured media from SR-uPA^+/0^ hearts [13.7 (6.4–26.3) versus 4.6 (2.3–7) pg/mg tissue/18 hrs, n = 5 mice per genotype, P<0.03, Figure 1A].

The increase in IL-6 protein levels was seen in cardiac tissue preceding fibrosis in SR-uPA^+/0^ mice, and IL-6 promotes fibroblast proliferation and collagen production. [31] Therefore, we hypothesized that IL-6 is an important proximal mediator of uPA-induced cardiac fibrosis. To test this hypothesis we generated SR-uPA^+/0^ with different *Il6* genotypes. At 15 weeks of age there was no difference in cardiac collagen accumulation between SR-uPA^+/0^ *Il6* ^−/−^, *Il6* ^+/−^ and *Il6* ^+/+^ mice respectively [9.8 (5.4–17.3) vs 10.1 (5.3–15.6) vs. 11 (9.3–14.7) percent picrosirius red area, n = 3–5 mice per genotype, Figure 1B]. All SR-uPA^+/0^ mice had significantly more collagen deposition than SR-uPA^0/0^ mice independent of *Il6* genotype [10.1 (6.5–15.9) vs. 1.7 (1.4–2.7), percent picrosirius red area, n = 11–13, P<0.001, representative images seen in Figure 1 D,E,F]. Heart weight to body weight ratios (HW/BW), and ventricular dimensions measured histologically in hearts arrested in diastole were not significantly different between genotypes (Table S1).

IL-6 is a potent macrophage chemokine that also may promote retention of macrophages in areas of injury (for review see [32]). Because macrophage accumulation is an important feature of uPA-induced cardiac fibrosis, we hypothesized that IL-6 is a mediator of cardiac macrophage accumulation in SR-uPA mice. To test this hypothesis we measured macrophage accumulation in SR-uPA^+/0^ mice with varying levels of IL-6. Absence of IL-6 did not reduce the density of macrophages in SR-uPA^+/0^ mice at 15 weeks of age [*Il6* ^−/−^24.4±13.6 vs. *Il6^+/+^*27.3±5.8 vs. *Il6^+/−^*38.5±30.2, Mac-3 positive cells per mm^2^, Figure 1C]. Consistent with our previous data in wild-type mice, NTG littermates had only rare Mac-3 positive cells in both the *Il6^+/−^* and *Il6^−/−^* backgrounds.

### uPA-expressing Macrophages are Polarized to Adopt an M2 Activation State

**Figure 2:**
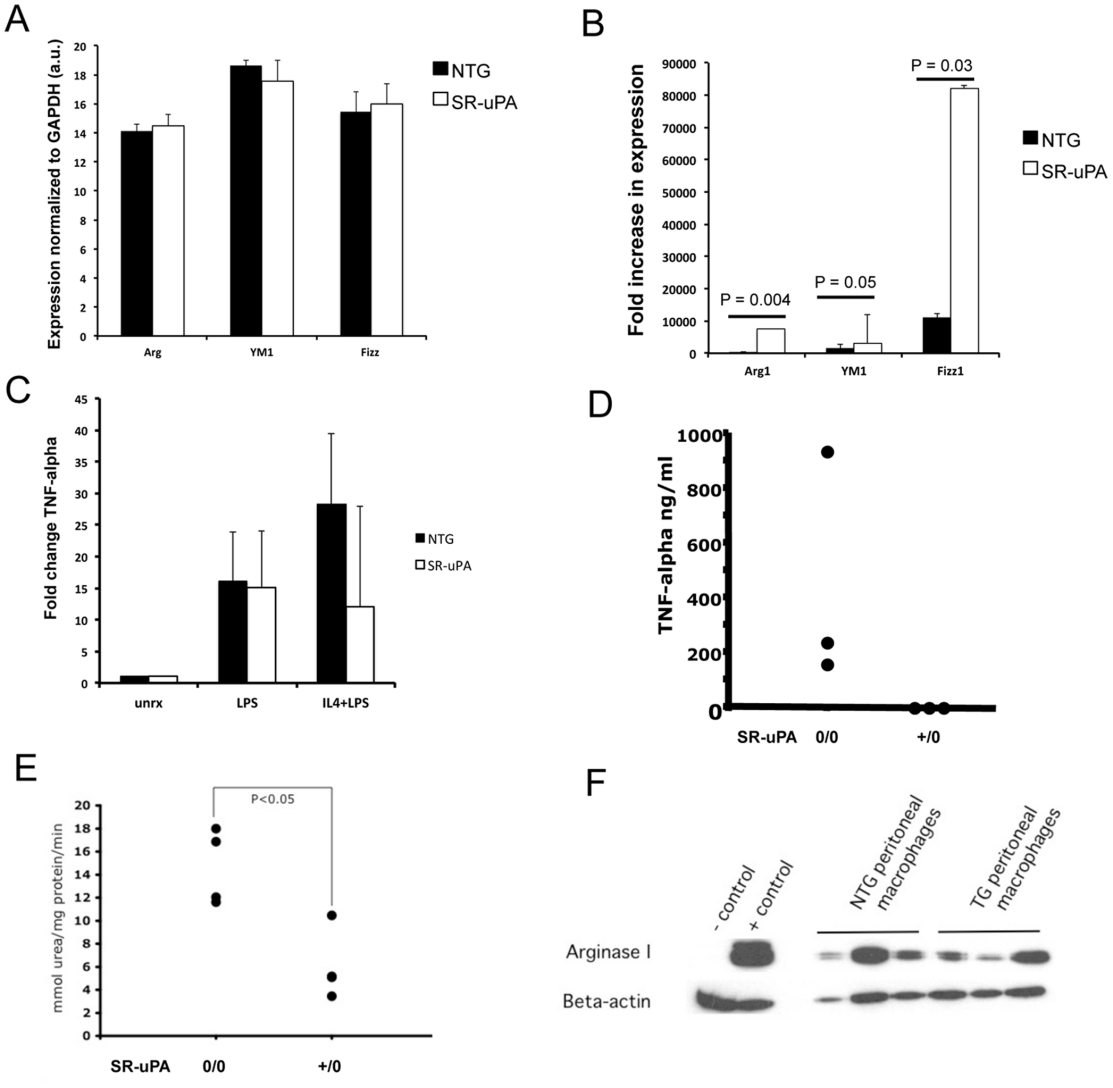
SR-uPA^+/0^ macrophages are polarized to adopt M2 phenotype. **A.** Expression of M2 genes measured by *qrtPCR* in SR-uPA^+/0^ and NTG macrophages. Columns represent the mean of arbitrary *C* units normalized to *Gapdh*, error bars are S.D., n = 5 mice per genotype. **B.** Increase in expression of markers of M2 activation in response to IL-4 (10 ng/ml) in SR-uPA^+/0^ and NTG bone marrow derived macrophages. Columns represent means and error bars represent S.E.M. *P* values are calculated by Mann-Whitney as data were non-parametric, n = 5–6 mice per genotype. **C.** Production of TNF-alpha protein after serial treatment with IL-4 followed by LPS in SR-uPA^+/0^ and NTG macrophages. Circles represent the mean of duplicate samples from individual mice, n  = 3 mice per genotype. **D.** Fold increase in expression of TNF-alpha mRNA after treatment with LPS or LPS+IL-4 in SR-uPA^+/0^ and NTG macrophages, n  = 4 mice per genotype. **E.** Arginase activity in conditioned media from SR-uPA^+/0^ and NTG macrophages. N = 4 mice per genotype **F.** Immunoblot for Arg1 in protein extract from SR-uPA^+/0^ and NTG macrophages.

M2 macrophages are associated with collagen accumulation *in vitro* and *in vivo* in non-cardiac models of injury and repair. Therefore, we tested the hypothesis that SR-uPA^+/0^ macrophages exhibit characteristics of M2 macrophages. To determine if SR-uPA^+/0^ macrophages were sensitized to adopt an M2 phenotype, we measured expression of the prototypic M2 genes, *Arg1*, *Retnla* (*Fizz1*) and *Chi313* (*Ym1*) at baseline and after stimulation with IL-4. There were no differences in baseline expression of these genes in macrophages from nontransgenic and SR-uPA^+/0^ littermates (14.1±0.1 vs. 14.5±0.15 for *Arg1*, 18.6±0.07 vs. 17.6±0.3 for *Ym1*, 15.4±0.28 vs. 16.0±0.27 for *Fizz1*; arbitrary *C* units normalized to *Gapdh*; n = 5 mice per genotype; Figure 2A). However, macrophages from SR-uPA^+/0^ mice had significantly greater fold-increases in expression of these genes after IL-4 treatment [450.6 (201–663) vs 4108 (2719–11565) for *Arg1*, 1608 (424–2605) vs 2872 (1968–4091) for *Ym1*, 61900 (22218–150709) vs 2097 (469–26256) for *Fizz1*; fold change versus untreated; n = 5–6 mice per genotype; Figure 2B). Because TGF-β1 is associated with M2 activation, directly induces fibrosis, and conditioned media from unstimulated SR-uPA^+/0^ macrophages induces fibrosis [33], we measured expression of *TGFB1* in unstimulated macrophages from SR-uPA^+/0^ and NTG mice. Consistent with our previous negative data on TGF-β1 protein and signaling in SR-uPA^+/0^ hearts, we found no increases in *TGFB1* expression in macrophages from SR-uPA^+/0^ vs. NTG littermates (11.3±1.2 vs 10.9±1.3, arbitrary *C* units normalized to 18 S, n  = 5 mice per genotype).

Some M2 macrophages have a decreased response to inflammatory stimuli. To test the hypothesis that SR-uPA^+/0^ macrophages had a decreased inflammatory response, we measured macrophage expression of TNF-α in response to LPS after pretreatment with IL-4. In comparison to macrophages from wild-type littermates, SR-uPA^+/0^ macrophages had an attenuated response to LPS after IL-4 pretreatment as measured by both protein levels and gene expression of TNF-α (Figure 2C, D).

Increased arginase activity is associated also with M2 mediated fibrosis [34]. Therefore, we measured arginase activity in isolated macrophages of SR-uPA^+/0^ mice and nontransgenic littermates using an established protocol [34]. Arginase activity was significantly elevated in macrophages of SR-uPA^+/0^ mice compared to nontransgenic littermates [14.4 (11.8–17.4) versus 5.1 (4.3–7.9) mmol urea/mg protein/min, n = 4, *P*  = 0.03, Figure 2E]. However, consistent with our expression data, SR-uPA^+/0^ macrophages did not produce increased amounts of Arg1 protein in comparison to NTG macrophages (0.48±0.16 versus 0.74±0.12 arbitrary units of Arg1 protein normalized to beta-actin Figure 2F). Therefore, we conclude that other M2 mechanisms such as down regulation of iNOS are contributing to increased arginase activity in SR-uPA macrophages.

### uPA Expressing Macrophages have Distinct Transcriptional Changes

**Figure 3:**
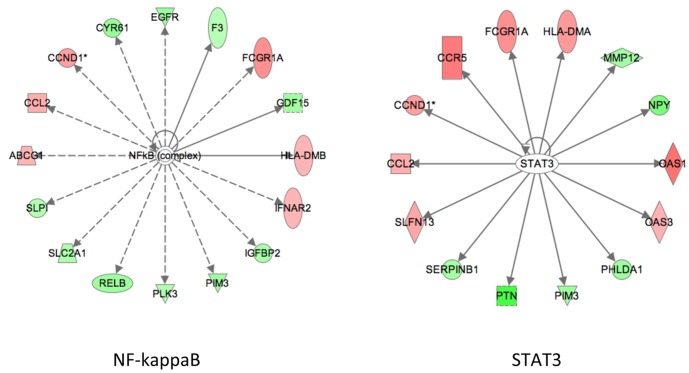
Transcriptome analysis of SR-uPA^+/0^ versus NTG macrophages. Interaction networks in which key molecules are transcriptionally differentially regulated in SR-uPA^+/0^ vs. NTG macrophages. Ingenuity Pathways Analysis (IPA) was used to make the network diagrams. In each network diagram, nodes represent specific molecules and transcriptional changes in SR-uPA^+/0^ vs. nontransgenic macrophages are indicated with blue or red circles (red = upregulated in SR-uPA^+/0^ vs. nontransgenic macrophages, green = downregulated).

To better understand the differential regulation of macrophage activation state in SR-uPA^+/0^ macrophages, we performed bead-array hybridization-based transcriptome profiling studies of bone marrow-derived macrophages from SR-uPA^+/0^ and non-transgenic control mice. Three female mice per genotype with identical parents were enrolled at 12 weeks of age. Using the Illumina 6-plex platform, we obtained high quality data on three SR-uPA^+/0^ and three nontransgenic mice. Analysis showed significant upregulation of two macrophage cell surface lectins associated with M2 polarization, *Mgl2* (3-fold, *P*<0.001), and *Mgl1* (1.7-fold, *P*<0.05). There was no evidence of upregulation of a well-described, pro-fibrotic factor, MMP or TGF–β1 dependent pathway. Furthermore, transcriptome analysis revealed a pattern previously described [35] in M2 polarized tumor associated macrophages, namely, upregulation of genes dependent on the STAT 3 pathways and down-regulation of factors dependent on NF-κB (Figure 3).

### uPA-expressing Macrophages Adapt M2 Phenotype after Migration into the Heart

**Figure 4:**
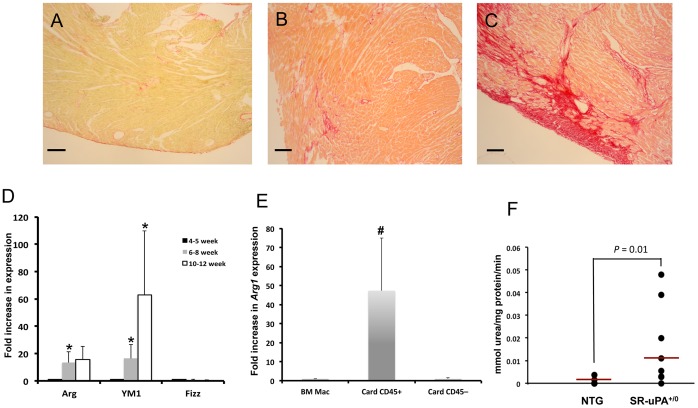
SR-uPA^+/0^ macrophages adopt full M2 phenotype in the heart. Picrosirius red stain for collagen in hearts from **A.** 5 weeks old, **B.** 7 weeks old, and **C.** 11 weeks old SR-uPA^+/0^ mice. Bars represent 100 µm. **D.** Fold increase in expression of *Arg1*, *Ym1* and *Fizz1* mRNA in CD-45+ cell fractions from hearts of SR-uPA^+/0^ mice at time-points of increasing fibrosis. Star (*) indicates significant *P* values versus 4–5 week time-point. N  = 4–7 mice per time-point. **E.** Comparison of *Arg1* expression from bone marrow macrophages, cardiac macrophages (CD45+) and cardiac CD45 negative fraction (CD45–). Pound (#) indicates significant *P* values versus other cell types, n  = 6–7 mice per condition. **F.** Arginase activity in cardiac explant conditioned media from SR-uPA^+/0^ and NTG hearts, n  = 7–8 mice per genotype.

Because fibrosis in SR-uPA^+/0^ mice is limited to the heart, we hypothesized that uPA-induced expression of M2 markers is potentiated after SR-uPA^+/0^ macrophages migrate to the heart. To test this hypothesis we examined hearts of SR-uPA^+/0^ mice at three time points: 4–5 weeks of age in which hearts have some macrophage accumulation without fibrosis; 7–8 weeks of age in which hearts have robust macrophage accumulation with minimal fibrosis; and 12 weeks of age in which both macrophage accumulation and fibrosis are robust (Figures 4A–C). We isolated RNA from bone marrow derived macrophages and cardiac-derived non-myocyte cells that were isolated and separated into CD45+ and CD45– fractions. We have previously shown that CD45+ cells in SR-uPA^+/0^ hearts are nearly all Mac3+ [23]. Quantitative PCR analysis showed significant increases in expression of *Arg1* [2.8 (1.1–26.5) at 6–8 weeks and 10.2 (1.2–36.2) at 10–12 weeks, fold change over 4–5 weeks, n = 4–7 mice per time point, *P*  = 0.035 for 6–8 weeks vs. 4–5 weeks] and *Chi313* (*Ym1*) [11.5 (4.1–31) at 6–8 weeks and 21.6 (13.4–133) at 10–12 weeks, fold change over 4–5 weeks, n = 4–7 mice per time point, P  = 0.004 for both time-points vs. 4–5 weeks] but not *Retnla* (*Fizz1*) [0.6 (0.6–0.8) at 6–8 weeks and 0.2 (0.04–0.6) at 10–12 weeks, fold change over 4–5 weeks, n = 6 mice per time point] over time in CD45+ fractions from SR-uPA^+/0^ hearts (Figure 4D, expressed as mean ±SEM for visual clarity).

Comparison of *Arg1* expression between BM macrophages, cardiac CD45+, and cardiac CD45– fractions, all harvested at 6–8 weeks of age, showed significant increases in *Arg1* expression only in the cardiac macrophage (CD45+) fraction [9.9 (3.8–94) for CD45+ cardiac cells versus 0.4 (0.3–1.2) for CD45– cardiac cells, fold increase over BMDM’s, n = 6–7 mice, *P*  = 0.002 and 0.001 for CD45+ vs. CD45– and BMDM repectively, Figure 4E]. To determine if increases in *Arg1* expression correlated with increased function, we tested arginase activity in hearts of SR-uPA^+/0^ and control mice. SR-uPA^+/0^ hearts had significantly more arginase activity at 5 weeks of age prior to the onset of fibrosis compared to nontransgenic littermates [0.1 (0.04–0.4) versus 0 (0–0.03) µmol urea/mg tissue/min, n = 7–8, *P*  = 0.01, Figure 4F]. Consistent with the upregulation in other M2 factors after migration to the heart, this increase in arginase activity was dramatically more than seen in isolated macrophages in Figure 2E.

We hypothesized that increases in M2 activation by cardiac macrophages were due increased concentrations of Th2 cytokines in hearts of SR-uPA^+/0^ mice. However, as described earlier, neither IL-4 nor IL-13 protein was detectable in conditioned media from heart extracts. To increase the sensitivity of detection of Th2 cytokines, we performed quantitative PCR for expression of *il4* and *il13* RNA in hearts of 8 weeks old mice. There was no difference in expression of *il13* in hearts of SR-uPA^+/0^ mice in comparison to NTG littermates [13.5±1.6 versus 13.9±1.2; arbitrary *C* units normalized to *Gapdh*, n = 3 mice per genotype]. We were unable to detect expression of *il4* from either genotype.

### uPA-expressing Macrophages Promote Collagen Expression in Cardiac but not Embryonic Fibroblasts

**Figure 5:**
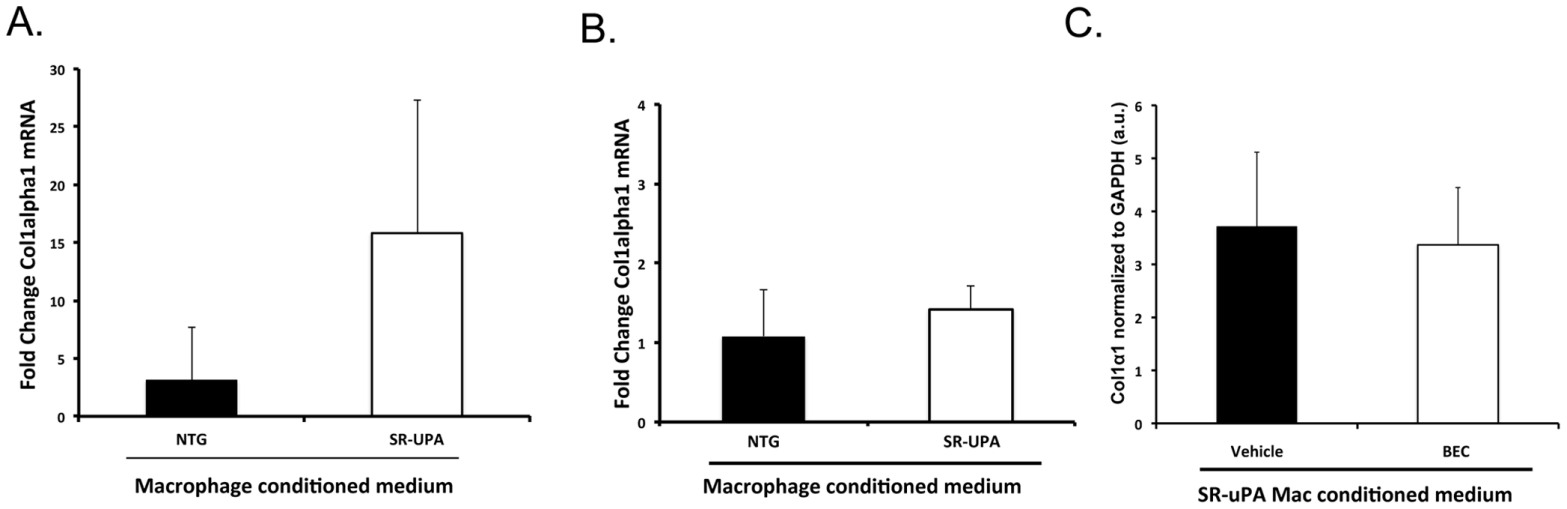
Pro-fibrotic effect of SR-uPA^+/0^ macrophages is limited to cardiac fibroblasts. Fold increase in expression in *Col1a1* mRNA in response to conditioned media from SR-uPA^+/0^ in comparison to NTG macrophages in **A.** isolated cardiac fibroblasts **B.** NIH-3T3 cells. Error bars represent S.D. N  = 4–6 mice per genotype. **C.** *Col1a1* expression (arbitrary *C* units normalized to *Gapdh*) in cardiac fibroblasts treated with conditioned media from SR-uPA^+/0^ macrophages treated with BEC or vehicle, N  = 4 mice per treatment, error bars represent S.D.

Our previous data indicate that in comparison to NTG macrophages, SR-uPA^+/0^ macrophages increase expression of *Col1α1* five-fold by cardiac fibroblasts [33]. Th2 cytokines secreted by M2 macrophages promote collagen production in both primary fibroblasts and fibroblast cell lines [36]; however, SR-uPA^+/0^ mice exhibit fibrosis specific to the heart. Therefore, we tested the hypothesis that macrophages expressing high levels of uPA promote collagen expression specific to cardiac fibroblasts. Serum-free conditioned media was collected from macrophages plated at similar density and placed on different fibroblast populations overnight. The following day fibroblast mRNA was isolated and expression of *Col1α1* was measured using qRT-PCR. Similar to our previous data with lower expressing SR-uPA^+/0^ macrophages, conditioned media from high expressing SR-uPA^+/0^ macrophages induced increased expression of *Col1α1* in isolated cardiac fibroblasts in comparison to conditioned media from nontransgenic littermates (15.2±11.5, fold increase versus nontransgenic conditioned media, n = 4 mice per genotype, Figure 5A). On the other hand, treatment of an embryonic fibroblast cell line (NIH-3T3 cells) with conditioned media from SR-uPA^+/0^ macrophages failed to increase *Col1α1* transcription in comparison to conditioned media from NTG littermates (1.4±0.6, fold increase versus NTG conditioned media, n = 6 mice per genotype, Figure 5B). In both cardiac and embryonic fibroblasts, TGF-β induced similar (∼2-fold) increases in *Col1α1* transcription. All values were normalized to *Gapdh* expression.

Because increased arginase activity was found in both isolated macrophages and heart, we tested the hypothesis that arginase is directly responsible for the increased collagen expression induced by SR-uPA^+/0^ macrophage conditioned media. To test this hypothesis, SR-uPA^+/0^ macrophages were treated for 24 hours with S-(2-boronoethyl)-L-cysteine (BEC), an inhibitor of arginase activity, and conditioned media harvested and placed on isolated cardiac macrophages. There was no difference in *Col1-α1* mRNA in cardiac fibroblasts treated with SR-uPA conditioned media from macrophages treated with vehicle or BEC (3.7±1.4 vs. 3.4±1.1 arbitrary units of qPCR *C* values of *Col1α1* expression normalized to *Gapdh* expression, n = 4, Figure 5C).

## Discussion

We have previously reported that over-expression of uPA in macrophages induces excess collagen deposition (fibrosis) limited to the heart [23], [24]. Moreover, plasmin/plasminogen activator activity is increased in human hearts and animal models in association with collagen deposition [37], [38]. Therefore, we sought to determine the mechanisms by which macrophage derived uPA promotes collagen deposition in the heart. Because, cardiac macrophage accumulation precedes fibrosis in SR-uPA^+/0^ mice and activation of pro-inflammatory (M1) macrophage cytokines is present in failing hearts [13], [14], we initially hypothesized that M1 macrophage activation is an important mediator of uPA-induced fibrosis. Here we present data that uPA expressing macrophages do not exhibit features of M1 (classical) macrophage activation. Even absence of IL-6–the single Th1 cytokine upregulated in the hearts of SR-uPA^+/0^ mice preceding fibrosis–did not attenuate uPA-induced cardiac fibrosis or macrophage accumulation. We found no differences in cardiac mass in *il6* ^−/−^ mice in comparison to wild type mice with or without the SR-uPA transgene. These data are in distinction to a report that IL-6 mice have dilated hearts [39] but consistent with a report of no differences in geometry in IL-6 null mice versus wild-type mice in response to injury [16]. From these data we conclude that classic pro-inflammatory macrophage activation pathways are not proximal mediators of uPA-induced fibrosis or macrophage accumulation in the heart.

Instead, our data support a role for a M2 macrophage activation pathway in uPA-induced cardiac fibrosis. We show here that SR-uPA^+/0^ macrophages are polarized to adapt an M2 phenotype in response to IL-4 and exhibited excess arginase activity, a feature of M2 activation [40]. Our findings that uPA augments polarization of macrophages to an M2 phenotype is consistent with a report that uPA-null mice (*plau* ^−/−^ mice) are susceptible to infections cleared by M2 activated macrophages [41]. Furthermore, IL-4 pretreatment significantly quenched the ability of SR-uPA^+/0^ macrophages induce inflammatory responses to LPS. A similar anti-inflammatory effect of the uPA/plasmin system on macrophage function has been observed in studies that demonstrated decreased levels of the macrophage marker *CD68* and the adipose inflammatory marker leptin, in mice deficient in PAI-1, the endogenous inhibitor of uPA [42]. Moreover, mice deficient in PAI-1 exhibit spontaneous cardiac macrophage accumulation and fibrosis late in life [23]. In sum, these data support a model in which excess plasmin activity (via increased uPA or decreased PAI-1) induces global changes in macrophages toward a M2 phenotype rather than inhibition of a particular inflammatory signaling pathway.

Our understanding of macrophage activation pathways is evolving at a rapid pace as variations on the M1/M2 polarization schema are elucidated in different disease states. A recent review proposed a model in which M1 and M2 phenotypes lie on a spectrum of macrophage activation states [11]. Applying this model to our data indicates that SR-uPA^+/0^ macrophages exhibit characteristics of both wound healing and immunoregulatory macrophage phenotypes. This blended phenotype has also been described in tumor-associated macrophages [TAM, for review see [43]]. Furthermore, our array data confirm that SR-uPA^+/0^ macrophages exhibit a similar pattern of transcriptional activation to TAM’s with decreased activity of NF-κB and increased activity of STAT3 [35]. Although uPA/plasmin activity is increased in many tumors [44], the role of this system in TAM development has not been, to our knowledge, explored.

It is intriguing that SR-uPA^+/0^ macrophages promote collagen deposition limited to the heart *in vivo*, and SR-uPA^+/0^ macrophages manifest their full M2/fibrotic phenotype only in the heart. Our data support several non-exclusive mechanisms for this specificity. First, the local cardiac environment may provide specific factors responsible for the full activation of SR-uPA^+/0^ macrophages to an M2 phenotype. Here we show that following migration into the heart, SR-uPA^+/0^ macrophages increase expression of the M2 marker *Arg1* in comparison to BMDM. The trigger for this activation is unknown. Our explant studies showed differences in protein levels or expression of the Th2 cytokines IL-4 or -13 in SR-uPA^+/0^ versus NTG hearts and our previous studies did not detect increased numbers of lymphocytes in hearts of SR-uPA^+/0^ mice [23]. However, we did confirm another report that *il13* is expressed in the mouse heart and may therefore contribute to fibrosis [45]. Because SR-uPA^+/0^ macrophages are sensitized to IL-4 stimulation (Figure 2B), it is possible that baseline cardiac levels on IL-13 are sufficient to activate SR-uPA^+/0^ macrophages to an M2 phenotype after migration. On the other hand, Dupasquier, et al noted that in the course of monocytes migrating to the skin to become Langerhans cells, M2 activation occurred independent of IL-4/13 signaling [46]. Crossing the SR-uPA^+/0^ mouse with the *Il4ra* knockout mouse would help resolve this mechanism, however, as the *Il4ra^−/−^* mouse strain is on a different background (BALB/c), such experiments would require extensive back crossing and breeding outside of the scope of this report.

Alternatively, the heart may be particularly sensitive to SR-uPA^+/0^ macrophages. In our previous work we noted that fibrosis is limited to the heart in SR-uPA^+/0^ mice [23], consistent with other studies showing an anti-fibrotic [47], [48] or neutral [49] effect of excess uPA in other organs. Our *in vitro* data support that the ability of SR-uPA^+/0^ macrophages to increase collagen production is limited to cardiac fibroblasts. Expression of *Col1α1* in response to conditioned media from SR-uPA^+/0^ macrophages is robustly upregulated in cardiac fibroblasts but not the NIH3T3 embryonic fibroblast cell line. This finding may explain why TAM’s with similar (but not identical) gene expression profiles rarely promote fibrosis in malignant tumors. Developmental studies support that tissues contain stem cells that contribute both to populations of specialized cells such as cardiomyocytes and “supportive” cells such as fibroblasts and vascular smooth muscle cells. These developmental differences in fibroblast populations may lead to differential sensitivity to pathologic stimuli in adulthood. Alternatively, quiescent fibroblast precursors may be the target of SR-uPA^+/0^ macrophages. Further studies to elucidate the role of these precursor populations in uPA-induced cardiac fibrosis are planned but outside the scope of this report.

The precise mechanisms by which SR-uPA^+/0^ macrophages induce collagen deposition remain unknown. TGF-β1 is reported as the classic M2 pro-fibrotic factor, due to its ability to directly stimulate fibroblast activation and expression of *Col1α1* [50], [51]. We have previously reported that neither TGF-β1 protein nor signaling is increased in hearts of SR-uPA^+/0^ mice prior to the onset of fibrosis [24]. Here we show that isolated SR-uPA^+/0^ macrophages do not express excess TGF-β1. In addition, array studies did not indicate increases in downstream products of TGF-β1 or increases in classic TGF-β1 transcription pathways. Although these array studies cannot completely rule out activation of all TGF-β1 signaling pathways in SR-uPA^+/0^ mice, our cumulative data support that classical signaling by TGF- β1 is not associated with uPA-induced cardiac fibrosis. Because arginase activity was upregulated in both isolated macrophages and hearts of SR-uPA mice, we hypothesized that arginase is a regulator of cardiac fibrosis in SR-uPA mice. Although there were no increases in Arg1 protein in SR-uPA^+/0^ macrophages, arginase activity can also be increased via decreases in iNOS signaling, and can promote fibrosis by increasing the availability of proline for collagen generation [52]. However, inhibition of arginase activity in isolated macrophages failed to diminish the pro-fibrotic capacity of these cells on cardiac fibroblasts. Therefore, our current data support the involvement of a novel cardiac specific pro-fibrotic factor.

In conclusion, we demonstrate that SR-uPA^+/0^ over-expressing macrophages exhibit a unique pattern of polarization to an M2/anti-inflammatory phenotype. These macrophages are associated with increased collagen in the heart and promote expression of *Col1α1* specifically in cardiac fibroblasts. Although IL-6 is upregulated in the hearts of SR-uPA^+/0^ mice, it does not appear to be a proximal mediator of uPA-induced cardiac fibrosis. Further studies to elucidate the mechanisms by which uPA expressing macrophages promote collagen deposition specific to the heart could result in significant advances in our therapeutic armamentarium for end stage heart disease.

## Supporting Information

Table S1 Cardiac Geometry in NTG and Transgenic Mice
(DOCX)
