# Supplementary material for: Urokinase Plasminogen Activator Induces Pro-Fibrotic/M2 Phenotype in Murine Cardiac Macrophages
Source: PLoS One. 2013 Mar 11;8(3):e57837. doi: 10.1371/journal.pone.0057837 (PMC3594198; doi:10.1371/journal.pone.0057837)
Supplement: Table S1 — Cardiac Geometry in NTG and Transgenic Mice (DOCX) [file pone.0057837.s001.docx]

| Genotype | HW/BW (mg/g) | Wall thickness (μm) | LVEDD (μm) |
| --- | --- | --- | --- |
| *il6*^-/-^ SR-uPA^+/0^ | 5.2±0.6 | 0.83±0.03 | 2.36±0.3 |
| *il6*^-/-^ SR-uPA^0/0^ | 5.4±0.4 | 0.89±0.08 | 2.46±0.3 |
| *il6*^+/+^ SR-uPA^+/0^ | 5.9±0.8 | 0.96±0.12 | 2.1±0.4 |
| *il6*^+/+^ SR-uPA^0/0^ | 4.9±0.4 | 0.94±0.03 | 2.6±0.17 |

**Supplemental Table 1:** Cardiac geometry in NTG and transgenic mice in presence and absence of IL-6. N= 3-9 mice per group. *P* is non-significant by one-way ANOVA.
